# Fudan Zenshin, Kyumeikyukyu ; ~Now JIN again~

**DOI:** 10.14789/jmj.JMJ22-0022-R

**Published:** 2022-08-01

**Authors:** HIROSHI TANAKA

**Affiliations:** 1 Department of Emergency and Disaster Medicine, Juntendo University Urayasu Hospital, Chiba, Japan; 1 Department of Emergency and Disaster Medicine, Juntendo University Urayasu Hospital, Chiba, Japan

**Keywords:** emergency medicine, pre-hospital emergency care, primary health care, intensive care, disaster medicine

## Abstract

I graduated from Osaka University in 1982 and joined the Department of Traumatology, Osaka University Medical School. Patients with severe injuries and illnesses were brought in every day. Staff brushed up their skills on site, taught each other, and engaged in friendly competition for research. We had many frustrating moments when we could not save lives. Since then, the needs of emergency medicine have changed, and the scope of practice of emergency physicians has expanded to include pre-hospital emergency care, primary health care, intensive care, and disaster medicine. I was transferred to Juntendo University Urayasu Hospital in September 2007. Soon after my assignment, Urayasu Hospital was in the spotlight due to the emergency hospitalization of the All-Japan soccer coach and the Chinese frozen dumpling incident. It is said that emergency medicine is a mirror of society. I myself have experienced many disasters and incidents. It has been 15 years since I was assigned to this hospital, and I have 62 colleagues at Urayasu Hospital. They have all acquired various medical specialties, and some are emergency medicine specialists. In 2019, we hosted the 47th Annual Meeting of the Japanese Association for Acute Medicine. The theme of the conference is “Fudan Zenshin, Kyumeikyukyu (constant advancement, emergency medical services)”. Emergency medical care is the starting point of “medicine” and is the ultimate source of life preservation for all citizens. We emergency physicians will continue to provide lifesaving medical care to patients without giving up until the very end, to keep the light of life from going out.

## Preface

I am pleased to announce that I will be retiring at the end of March 2022. I would like to express my sincere gratitude to Chairman Hideoki Ogawa and the many others who have guided and supported me throughout the years.

## Before moving to Juntendo University

I graduated from Osaka University in 1982 and joined the Department of Traumatology, Osaka University Medical School because of my admiration for the work “Black Jack” by Osamu Tezuka, my senior in high school and college. At that time, patients with severe trauma, burns, poisoning, sepsis, and other serious illnesses were brought in every day. Staff brushed up their skills on site, taught each other, and engaged in friendly competition for research. We had many frustrating moments when we could not save lives. My mentor, Professor Tsuyoshi Sugimoto, often told me, “general surgeons should be ashamed of intraoperative death, and emergency surgeons should be ashamed when they miss the timing of surgery and let a patient die.” Currently, the needs of emergency medicine have changed, and the scope of practice of emergency physicians has expanded to include pre-hospital emergency care, primary emergency care, intensive care, and the field of disaster medicine, medical control in the region.

## Frozen poisonous dumpling incident made in China

**Figure 1:**
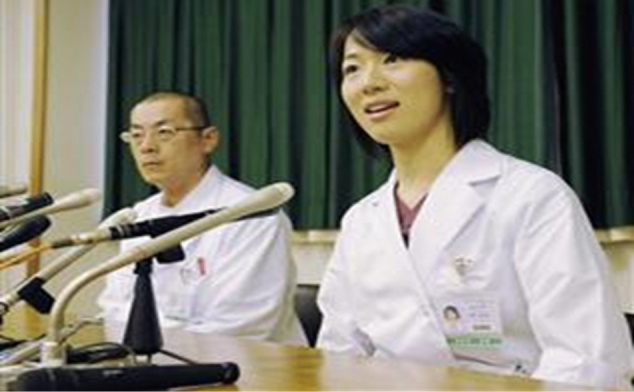
I and Dr Yuka Sumi got interviewed from a lot of media after the patient got well. Dr Sumi who is a member of us, and now she is working at the World Health Organization.

I was transferred to Juntendo University Urayasu Hospital in September 2007. Soon after my assignment, Urayasu Hospital was in the spotlight due to the emergency hospitalization of the All-Japan soccer coach and the Chinese frozen dumpling incident^1)^. An outbreak of food poisoning that affected at least ten people in various regions of Japan was traced to exposure to Chinese dumpling contaminated with the organophosphate insecticide Methamidophos. We experienced the most serious case, a five years old girl, who suffered coma. She presented with features of cholinergic overactivity and her serum cholinesterase activity was very low. We started intravenous treatment with pralidoxime iodide, atropine sulfate, and midazolam. Her symptoms improved gradually and she was discharged on day 25 without any sequelae. I and Dr Yuka Sumi got interviewed from a lot of media after her illness recovered (Figure 1). Dr Sumi, a member of us, is now working at the World Health Organization.

## Emergency medicine is a mirror of society.

**Figure 2:**
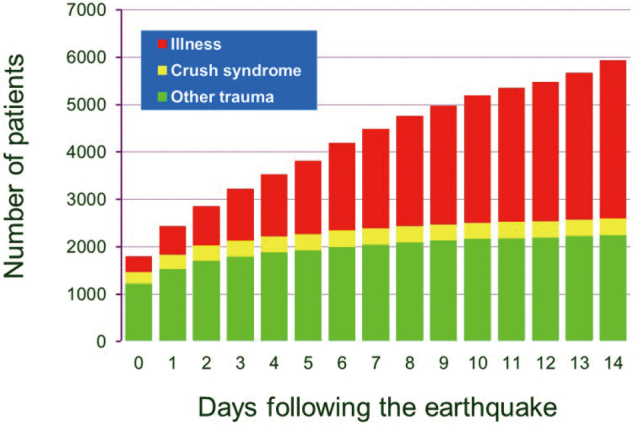
Cumulative patient census graph of all patients admitted to surveyed hospitals: green box, injury without crush syndrome; yellow box, crush syndrome; red box, illness.

It is said that emergency medicine is a mirror of society. I myself have experienced many disasters and incidents (Table 1). In Osaka, I provided medical care for the Great Hanshin-Awaji Earthquake^2)^, the hemolytic uremic syndrome by O157 mass food poisoning, the Ikeda Elementary School child murder cases, and the JR Fukuchiyama train derailment accident. A catastrophic earthquake registering 7.2 on the Richter scale hit the southern part of Hyogo Prefecture including the city of Kobe, at 5:46 AM on January 17, 1995. The earthquake, subsequently knows as “Hanshin-Awaji,” caused approximately 5,500 deaths and 41,000 injuries. Most of the dead were crushed or suffocated in collapsed dwellings. The number of partially destroyed dwellings reached approximately 100,000, and the number of those completely collapsed reached 93,000. Seven thousand one hundred dwellings burned down. We demonstrated morbidity and mortality of hospitalized patients within 14-days after the earthquake. Of the total 6,107 patients admitted to the 95 surveyed hospitals, 2,718 were injury patients, comprising 372 crush syndrome patients and 2,346 patients with other trauma. A total of 3,389 patients presented with illness. The mortality rates were 13.4% (50/372), 5.5% (128/2,346), and 10.3% (349/3,389) in crush syndrome, other trauma, and illness, respectively (Table 2). All patients admitted to surveyed hospitals are represented by the graph in Figure 2. Approximately 75% of trauma patients were hospitalized during the first 3 days. In contrast, the number of patients hospitalized for illness continued to increase during the entire 15-day period.

**Table 1:** Recent mass disaster and incident in Japan (2000~

VX nerve agent cases (1994)
the Great Hanshin-Awaji Earthquake (1995)
A series of sarin cases (1995)
O157 mass food poisoning (1995)
Wakayama Curry Incident (1998)
The Ikeda Elementary School child murder case (2001)
the JR Fukuchiyama train derailment accident (2005)
The Chinese frozen dumpling incident (2008)
The Akihabara indiscriminate murder case (2008)
The Great East Japan Earthquake (2011)
Mt Ontake eruption (2014)
Kinugawa flood (2015)
Kumamoto earthquake (2016)
Arson attack on an anime station in Kyoto (2019)
COVID-19 pandemic (2020)

**Table 2:** Patient Cllasfications and the Number of Related Deaths

	No. of Patients	No. of Deaths (%)
Crush syndrome	372	50 (13.4)
Other trauma	2346	128 (5.5)
Illness	3389	349 (10.3)
Total	6107	527 (8.6)

## The first case of the efficacy of steroid treatment for COVID-19.

**Figure 3:**
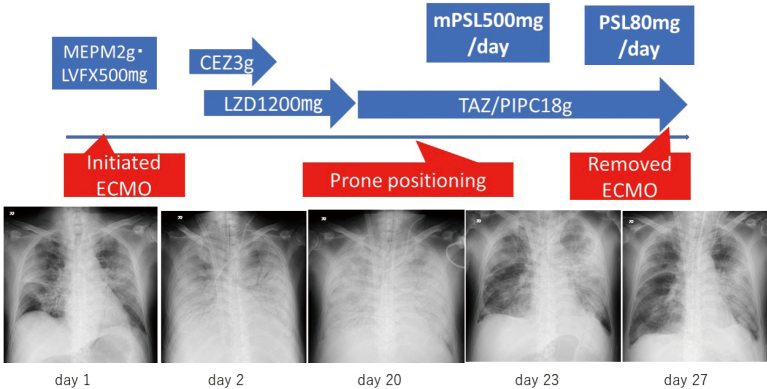
Chest X-ray images in time series of a 67-year old man during hospitalization for severe COVID-19-induced acute respiratory distress syndrome. Chest X-ray on day 1 shows bilateral basal consolidation with periphearal ground-glass opacity. Chest X-ray on day 2 shows bilateral pulmonary infiltrate mainly in hilar region. Chest X-ray on day 20 shows bilateral diffuse pulmonary infiltraes with air bronchogram. Chest X-ray on day 23 shows improved bilateral lung opacity (mainly in the right lobe of lungs). Chest X-ray on day 27 (extracorporeal membrane oxygenation removed).

After my transfer, I also experienced many disasters and incidents, including the Great East Japan Earthquake, and the coronavirus disease (COVID-19) pandemic, with which we are still battling. We reported the first case of the efficacy of steroid treatment for COVID-19^3)^. A 67-year old man was transported to our hospital due to impaired consciousness and respiratory failure. After admission, tracheal aspirate of the patient was harvested, and it tested positive for severe acute respiratory syndrome coronavirus 2 nucleic acid. He required veno-venous extracorporeal membrane oxygenation (V-V ECMO) to sustain his oxygenation. However, his respiratory failure did not improve for 20 days. On day 20 of admission, we started to use i.v. steroid therapy. On day 23, lung opacity on the chest X-ray cleared and removed ECMO on day 27. We are successfully tapering steroids without serious adverse events, and he was removed from the ventilator on day 51 (Figure 3).

## Our various research areas (from clinical research to basic one)

It has been 15 years since I was assigned to this hospital, and I have 62 colleagues at Urayasu Hospital. They have all acquired various medical specialties, and some are emergency medicine specialists. Eighteen have earned doctoral degrees in medicine and many have studied abroad at Harvard University and the Feinstein Institute in New York.

### -Clinical research-

Our research areas are diverse (Table 3). In clinical research, studies on the emergency room included triage by rapid lactate measurement, relationship with dizziness and heart rate variability, while registry studies in pediatric emergencies included prognostic comparisons of pediatric severe trauma, and epidemiological studies of critically ill pediatric patients. Clinical research in the field of intensive care includes systemic review on hyperosmotic infusion in patients with severe head trauma and the development of a prognostic model for patients with cardiopulmonary arrest^4)^.

**Table 3:** Our various research areas

Clinical research
Studies on ER science
Triage by rapid lactate measurement
Dizziness and heart rate variability
Registry studies in pediatric emergencies
Prognostic comparison of pediatric severe trauma
Epidemiological studies of critical ill pediatric pateient
Intensive care field
Systemic review on hyperosmotic infusion in severe head injury
Prognostic model for patients with cardiopulmonary arrest
Role of complement system in patients with multiple organ failure (KAKENHI Grant number 19H03764)
Disaster and Prehospital research
BCPs for disasters
Studies on securing intensive care staff during a pandemic
Studies on water supply in the earthquake
Effectiveness rapid response car
Establishment of an emergency medical care system in large theme park
Effectiveness of electronic triage during disasters (CREST)
Basic research
Neutrophil phenotype studies in sepsis
Dynamic of aged neutrophils
ICAM-1 positive neutrophils and NETs
Identification of low-density neutrophil
ATP targeting studies
Fluorescence imaging of ATP on the surface of neutrophils and in mitochondria
Neutrophil signaling transduction
Joint research course
Emergency AI Color Image Information Standardization Course

### -Disaster medicine and pre-hospital emergency medicine-

Studies in the field of disaster medicine and pre- hospital emergency medicine involves those on the establishment of business continuing plans (BCPs) for disasters. Other studies include research on the effectiveness of Rapid Response Car^5)^, the establishment of an emergency medical care system in large theme parks, and research on the effectiveness of electronic triage during disasters, which was adopted by CREST.

### -Basic research (neutrophil phenotype and ATP)-

**Figure 4:**
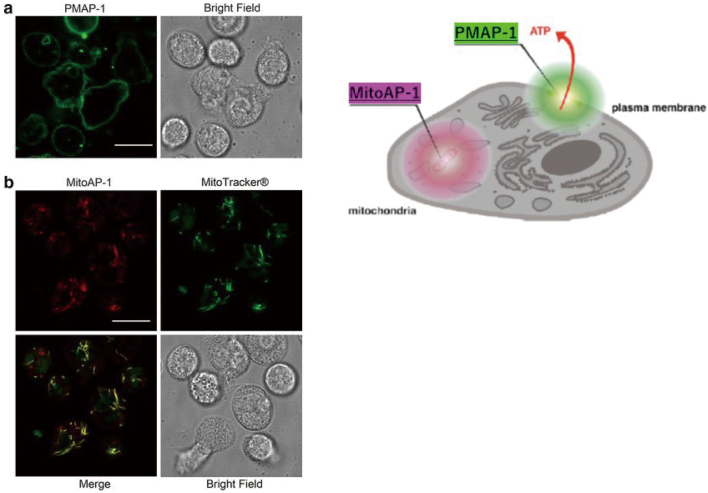
Confocal micrographs of human polymorphonuclear neutrophils (PMNs) stained with PMAP-1 and MitoAP-1. a ATP on the plasma membrane was stained with PMAP-1. The *bright green* fluorescence observed on the plasma membrane of PMNs resulted from PMAP-1 conjugation to ATP (×100 oil objective, NA 1.4). *Scale bar* 10 μm. b ATP in the mitochondria was stained with MitoAP-1 (*red*) and MitoTracker^®^ Green FM (*green*), and they colocalized well (×100 oil objective, NA 1.4). Scale *bar* 10 μm

**Figure 5:**
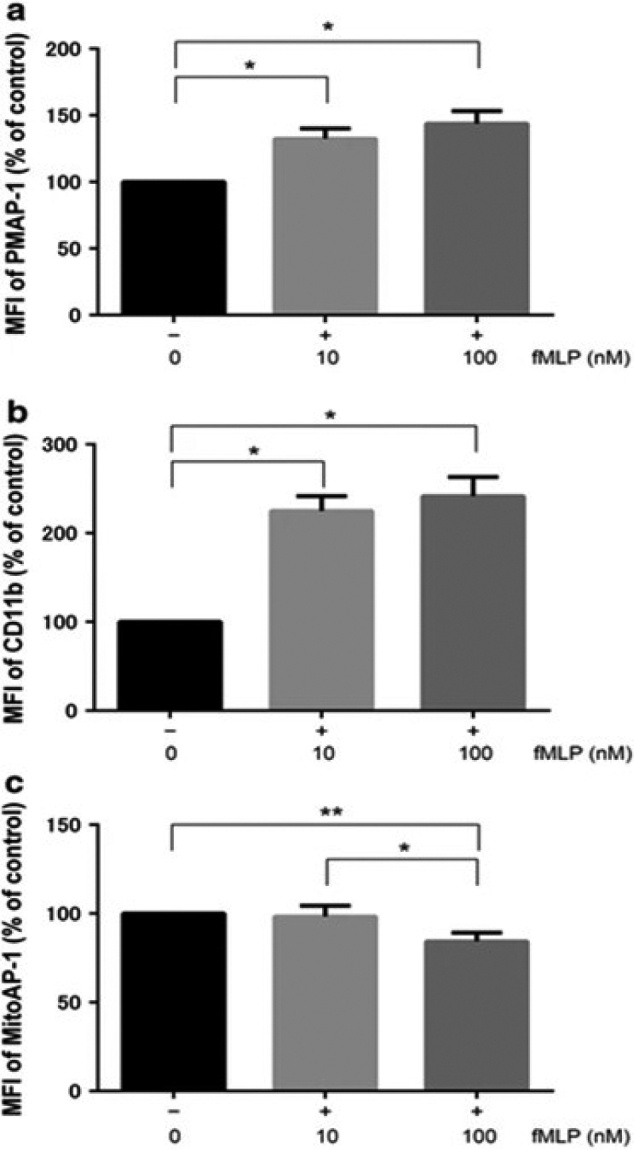
ATP level and CD11b expression after fMLP stimulation in healthy control subjects. a Changes in the mean fluorescence intensity (MFI) of PMAP-1 after stimulation with the indicated fMLP concentrations; MFI values were normalized to those of controls (no fMLP) (n = 8 per group). The data shown are the mean ± SEM, and groups were compared with one-way ANOVA and Tukey’s post hoc test (*p < 0.01). b Changes in CD11b expression of polymorphonuclear neutrophils after fMLP stimulation; expression levels were normalized to those of controls (no fMLP) (n = 8 per group). The data shown are the mean ± SEM, and groups were compared with one-way ANOVA with Tukey’s post hoc test (*p < 0.01). c Changes in MFI of MitoAP-1 after stimulation with the indicated fMLP concentrations; MFI values were normalized to those of controls (no fMLP) (n = 8 per group). The data shown are the mean ± SEM, and groups were compared with one-way ANOVA with Tukey’s post hoc test (*p < 0.01, **p < 0.05)

Basic research includes neutrophil phenotype studies during invasion (e.g., during sepsis) and studies targeting ATP. The former includes the dynamics of aged neutrophils, ICAM-1 positive neutrophils and NETs, and identification of low- density neutrophil subsets. In the research on neutrophil ATP, we conducted studies on fluorescence imaging of ATP on the surface of neutrophils and in mitochondria, and neutrophil signaling transduction (Figure 4)^6)^. Stimulation of the formyl peptide receptor was previously found to cause ATP release from PMNs through maxi-anion channels and PANX1 hemichannels. To confirm this finding, the median fluorescence intensity (MFI) of PMAP-1 on the plasma membrane of healthy control (HC) PMNs was quantified by flow cytometry after stimulation with the indicated concentrations of fMLP (Figure 5a). The MFI of PMAP-1 following fMLP stimulation increased in a dose-dependent manner. CD11b expression on the plasma membrane of PMNs was also measured as a marker of PMN activation. Cell surface CD11b expression in PMNs stimulated with fMLP also increased in a dose-dependent manner (Figure 5b). Mitochondria are often referred to as the powerhouse of the cells, as they generate ATP by oxidative phosphorylation. To our knowledge, changes in mitochondrial ATP levels in PMNs have not been reported. The MFI of MitoAP-1 following fMLP stimulation decreased significantly in a dose-dependent manner (Figure 5c). The MFIs of PMAP-1 and MitoAP-1 in sepsis patients were evaluated by flow cytometry within 24 h after diagnosis of sepsis (days 0-1). Both values were significantly higher than those of the HC group (data not shown). CD11b expression on the plasma membrane was also upregulated in sepsis patients. Activated PMNs in sepsis patients appeared to cause a burst of extracellular ATP release and to increase ATP synthesis by oxidative phosphorylation in the mitochondria. The same experiments were conducted at days 3-4 as the patients' clinical conditions improved, which revealed that the MFIs of PMAP-1 and CD11b expression decreased significantly compared to those at days 0-1, whereas a high MitoAP-1 MFI was maintained. Namely, the temporal changes of ATP release and CD11b expression on the plasma membrane were similar, but ATP production showed distinct behavior in the mitochondria.

### -Complement system in multiple organ failure-

Recently we are trying to elucidate the of complement system in patients with multiple organ failure by the grant for Japan Society for the Promotion of Science (JSPSI, KAKENHI Grant number 19H03764). Sepsis is a life-threatening emergency that occurs when the human body reacts in an extreme way to an infection, triggering a chain reaction that exacerbates the patient's condition. Almost any type of infection can lead to sepsis, although the most common infections typically start in the lung, urinary tract, skin or gastrointestinal tract. One of the main problems associated with sepsis is the multiple organ failure or dysfunction that it can lead to - it is this which most often leads to complication resulting in death. However, despite much research into this subject, the actual causes for multiple organ failure from sepsis (as well as severe trauma, burns and heat stroke) are not entirely clear. We have turned our attention to trying to ascertain the exact reasons why multiple organ failure occurs in sepsis patients. We have developed a hypothesis that one of the causes for multiple organ failure in sepsis patients is complement activation. The complement system is a term denoting a series of more than 20 proteins that circulate in the blood and tissue fluids. Complement causes the killing of bacteria and the recycling of dead cells in the body and is essential to an effective immune response in the human body. Complement activation occurs during a range of alien invasions, such as viral infection, allergy, severe trauma, heat stroke and sepsis. In recent years, it has become clear that excessive complement activation can cause thrombotic microangiopathy (TMA), which is a condition that can lead to organ damage. Knowing this, we set about working to understand the extent to which complement activation is involved in multiple organ failure during biological infection. It is known that when the protein complement component 3 (C3) is activated by the immune system in response to infection, foreign objects or external stimulus, it becomes C3a and C3b, the latter of which binds to the surface of the cell membranes of microorganisms and reacts with factor B and factor D to form C3 converting enzymes. Moreover, when this particular pathway weakens the function of complement regulators in the body, it becomes amplified and becomes C5a and C5b which leads to the development of a variety of pathological conditions. We are therefore working on aspects of this knowledge with a view to determining whether uncontrolled complement activation occurs during biological infection and whether TMA is triggered, eventually leading to multiple organ failure. To achieve their aims, we have investigated the suppression of multiple organ failure in both clinical and basic research by studying the quantitative evaluation of the complement activation, its relationship with TMA, the relationship between complement activation and leukocyte/platelet and the control of complement activity. To perform the experiments, we used tools that are common to immunology, such as ELISA (enzymelinked immunosorbent assay) and FACS (flow cytometry)^7)^.

### -A joint research course (Emergency AI Color Image Information Standardization Course)-

Additionally, a joint research course (Emergency AI Color Image Information Standardization Course) was established with Toppan Printing Co. Ltd since last year. This study pursues the authenticity of color image data in the field of emergency medicine. Our goal is to build a platform for providing medical image information and to apply it clinically to emergency medical care settings using image information standardized by numerical color information.

## The 47^th^ Annual Meeting of the Japanese Association for Acute Medicine

**Figure 6:**
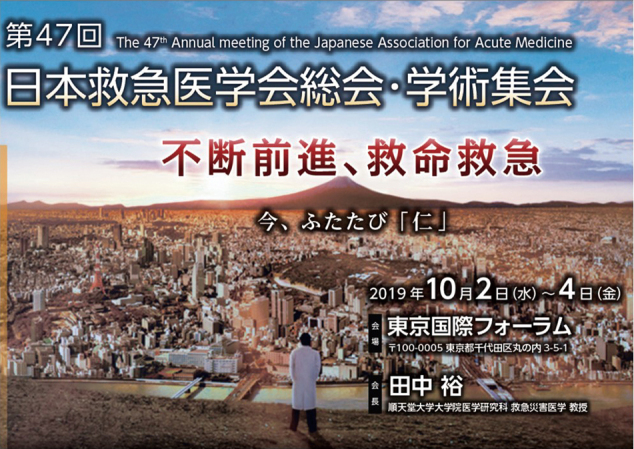
The 47^th^ annual meeting of the Japanese Association for Acute Medicine, held at Octorber 2 to 4, 2019 in Tokyo.

In 2019, we hosted the 47th Annual Meeting of the Japanese Association for Acute Medicine (Figure 6). The theme of the conference is “Fudan Zenshin, Kyumeikyukyu; Now JIN again.”. We have been continuously moving forward in acute medicine and critical care (Fudan Zenshin, Kyumeikyukyu), with Juntendo spirit JIN (humanity) that is now required again. Emergency medical care is the starting point of “medicine” and is the ultimate source of life preservation for all citizens. We emergency physicians will continue to provide lifesaving medical care to patients without giving up until the very end, to keep the light of life from going out.

## Conclusion

**Figure 7:**
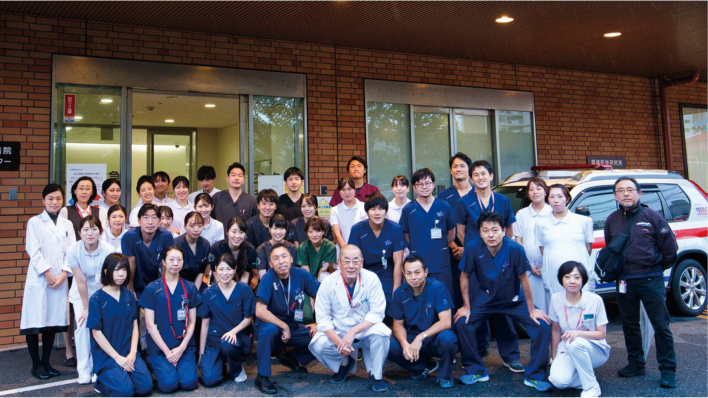
Current medical staff in the Department of Emergency and Disaster Medicine, Urayasu Hospital.

Finally, I would like to thank all the medical staff and my family for their support. I would like to conclude my retirement address by wishing Juntendo's continued growth and good health to all those who are a part of Juntendo (Figure 7).

## Funding

No funding was received.

## Author contributions

HT. wrote the manuscript.

## Conflicts of interest statement

The author has no conflict of interest to disclose.
